# Effect of Grandmaternal Smoking on Body Size and Proportions at Birth

**DOI:** 10.3390/ijerph18094985

**Published:** 2021-05-07

**Authors:** Isabell Katharina Rumrich, Otto Hänninen, Matti Viluksela, Kirsi Vähäkangas

**Affiliations:** 1 Department of Health Security, Finnish Institute for Health and Welfare (THL), P.O. Box 95, 70701 Kuopio, Finland; otto.hanninen@thl.fi (O.H.); matti.viluksela@uef.fi (M.V.); 2 School of Pharmacy/Toxicology, Faculty of Health Sciences, University of Eastern Finland (UEF), P.O. Box 1627, 70211 Kuopio, Finland; kirsi.vahakangas@uef.fi

**Keywords:** pregnancy, tobacco, smoking, maternal, grandmaternal, birth outcome, birth weight, register research, intergenerational

## Abstract

Many long-term adverse effects of smoking during pregnancy are known. Increasingly, adverse effects in the grandchild after grandmaternal smoking during pregnancy are reported. We explored this in a birth cohort of 24,000 grandmother–mother–child triads identified from the Finnish Medical Birth Register in 1991–2016. Multiple logistic regression was used to analyze the association between any smoking during pregnancy by both grandmother and mother, or only grandmother or mother on adverse birth outcomes. No smoking by neither grandmother nor mother was used as the reference. As endpoints, preterm birth, low birth weight, small for gestational age (birth weight, birth length, head circumference), and body proportionality (low ponderal index, high brain-to-body ratio, high head-to-length ratio) were included. Smoking by both grandmother and mother was consistently associated with higher risks than smoking only by the mother. Birth length and weight were especially sensitive to (grand)maternal smoking. In conclusion, the combined effect of grandmaternal and maternal smoking is associated with higher risks than only maternal smoking.

## 1. Introduction

Although smoking during pregnancy is decreasing in Finland (about 15% in 2016, less than 11% in 2019), the youngest mothers still smoke at alarming frequency: almost 35% in ≤ 19 year old pregnant women, and about 21% in 20–24 year old pregnant women in 2019 [1].

Despite the long tradition of smoking research, the understanding of intergenerational effects of maternal smoking is limited. The evidence of adverse effects by grandmaternal smoking on birth weight is inconclusive. While Rillamas-Sun and coworkers [2] report no clear effects, other studies suggest an increase in birth weight [3] and higher BMI or waist circumference [4,5]. Others have found similar associations only for the paternal line [6]. In contradiction, Golding and colleagues [7] report increased lean body mass in grandsons and reduced body mass in granddaughters. Previous studies have reported an association between grandmother’s smoking and increased asthma risk in the child [8,9,10]. Furthermore, an association of grandmaternal smoking with increased risk for autism in the grandchild has been reported [11]. The adverse effects of maternal smoking on the developing child are well documented [12,13], with animal studies implicating effects even in future generations [14,15]. In humans, asthma in children is associated with grandmaternal smoking [8]. With increasing understanding of epigenetic modifications as important mediators of inter-generational effects (for a review see, e.g., [16]), interest into the effects of grand-parental exposures is growing. Animal studies have been able to pinpoint specific epigenetic changes related to modified brain function by nicotine [17]. Grandmaternal smoking exposes the future mother during her prenatal development, most probably leading to epigenetic changes in the developing germ cells and thus affecting future children, as animal studies indicate [17,18].

Our aim was to explore the potential effects of grandmaternal smoking on the risk for adverse birth outcomes. Our objectives were to investigate the potential effects of (i) grandmaternal or maternal smoking separately, and (ii) combined grandmaternal and maternal smoking on preterm birth, low birth weight, small body size for gestational age and changes in body proportions in newborns.

## 2. Materials and Methods

### 2.1. Study Design

This study is a register-based study utilizing the MATEX birth cohort, which was retrieved from the Finnish Medical Birth Register (MBR) [19]. In the MBR, all births in Finland are recorded after the 22nd gestational week or births of a baby with at least 500 g birth weight. Information is collected on standardized forms by nurses and midwives during antenatal care visits of the mother and during and after delivery of the baby. Shortly, in the MBR, information is available on the mother and her health, pregnancy complications, delivery characteristics, and the newborn’s characteristics and health. The MATEX cohort is described in more detail elsewhere [20]. Within this cohort, roughly 24,000 triads of grandmother-mother-child were identified. All singleton births without major birth anomalies, for which smoking information of mother and grandmother were available, were included. The newborns included were born between 2005 and 2016, and the mothers were born between 1991 and 2006.

### 2.2. Exposure

The smoking category of grandmother and mother was defined based on the self-reported smoking status recorded in the MBR. In the MBR, the smoking categories are “no smoking”, “quit smoking during first trimester”, “continued smoking after 1st trimester”, and “no information”. The latest group has been excluded from the analyses. To increase statistical power, the categories of “quit smoking during the first trimester” and “continued smoking after the first trimester” have been combined to “any smoking during pregnancy” (Table 1).

### 2.3. Outcomes

In this work, we consider four types of outcomes (Supplementary Materials, Table S1). Firstly, preterm birth defined based on a gestational age of less than 37 completed weeks gestation. Secondly, low birth weight defined as weight at birth below 2500 g.

Thirdly, we included endpoints evaluating the body size for the corresponding gestational age at birth. Within this group we defined a non-specific summary endpoint consisting of all body dimensions (birth weight or body length at birth or head circumference at birth) below the 10th percentile in the Finnish reference growth chart [21]. In addition, we analyzed separately the following endpoints: low birth weight for gestational age, short body length for gestational age, and small head circumference for gestational age.

As a fourth group of endpoints, we included indicators for body proportions, such as ponderal index, brain-to-body ratio, and head-to-length ratio. Ponderal index was calculated using birth weight and body length Equation (1). It was categorized as normal (10–90th percentile of the study population, used as the reference) and low (<10th percentile). Newborns above the 90th percentile were excluded.

(1) $$\mathrm{Ponderal}\;\mathrm{Index}=100\times \frac{\mathrm{birth}\;\mathrm{weight}\;\left[g\right]}{\mathrm{body}\;\mathrm{length}\;{\left[\mathrm{cm}\right]}^{3}}$$

Brain-to-body ratio was calculated based on head circumference and birth weight Equation (2). It was categorized as high (>90th percentile of the study population) and normal (10–90th percentile, reference). Newborns below the 10th percentile were excluded.

(2) $$\mathrm{Brain}\;–\mathrm{to}–\mathrm{Body}\;\mathrm{Ratio}=100\times \frac{0.037\times \mathrm{head}\;\mathrm{circumference}\;\left[\mathrm{cm}\right]{\;}^{2.57}}{\mathrm{birth}\;\mathrm{weight}\;\left[g\right]}$$

The nominator of the formula is the estimation of the brain weight according to the National Institute of Neurological and Communicative Disorders and Stroke’s Collaborative Perinatal Project [22].

Head-to-length ratio was calculated using head circumference and body length Equation (3). It was categorized normal (10–90th percentile of the study population, reference) and high (>90th percentile). Newborns below the 10th percentile were excluded.

(3) $$\mathrm{Head}\;–\mathrm{to}–\mathrm{Length}\;\mathrm{Ratio}=\frac{\mathrm{head}\;\mathrm{circumference}\;\left[\mathrm{cm}\right]}{\mathrm{body}\;\mathrm{length}\;\left[\mathrm{cm}\right]}$$

### 2.4. Covariates

Maternal and grandmaternal age were used in years as continuous variables. Gestational age was used in the low birth weight regression analyses as continuous variable in weeks. Parity was defined as nulli- or multiparous. The Finnish national classification of occupations was used to categorize the socioeconomic status as upper white collar (upper level employees with administrative, managerial, professional, and related occupations), lower white collar (lower level employees with administrative and clerical occupations), blue collar (manual workers), and others (famers, self-employed, students, pensioners, no information). In addition, a category of “missing information” was added [23]. Maternal low birth weight and maternal preterm birth were defined as described above in the Endpoint section. Maternal co-morbidities were defined based on ICD-10 codes as a binary variable coded as “yes” and “no”. Co-morbidity was categorized as “yes” if one or more of the following diagnostic codes were recorded in the MBR: O10 (Pre-existing hypertension complicating pregnancy childbirth and the puerperium), O11 (Pre-existing hypertension with pre-eclampsia), O12 (Gestational [pregnancy-induced] edema and proteinuria without hypertension), O13 (Gestational [pregnancy-induced] hypertension without significant proteinuria), O14 (Pre-eclampsia), O16 (Unspecified maternal hypertension), and O24 (Diabetes mellitus in pregnancy, childbirth, and the puerperium).

### 2.5. Statistical Analysis

A multivariate logistic regression was used to estimate odds ratios (OR) and 95% confidence intervals. In all analyses, the reference group included triads with neither grandmother nor mother smoking during pregnancy. The regression models where adjusted for potential confounders (Table 2). Adjustment models were developed based on a combination of previously published models and data availability in the MATEX cohort.

The data were analyzed using R Statistical software (version 3.6.3 (29 February 2020)-“Holding the Windsock”, R Core Team, Vienna, Austria) [24]. The statistical power of the study has been estimated using R function epi.sscohortc in package epiR (version 2.0.19) [25] with a power of 90% and 95% confidence interval (Table 3). The regression analysis was conducted using the R base function glm with binomial family and logit link.

### 2.6. Ethics Approval and Register Data Permit

In accordance with the Finnish Medical Research Act (1999/488), the MATEX study including the birth cohort identified from the Medical Birth Register has been evaluated and approved by the official ethics committee of the Northern Ostrobothnia Hospital District (EETTMK 44/2016; issued 18 April 2016). The right to use register data held by the Finnish Institute for Health and Welfare was granted under the document number THL/838/6.02.00/2016 (issued 22 June 2016) and extended under document number THL/984/6.02.00/2020 (issued 17 March 2020). Due to the full register-based design of the study, no informed consent was required from the study participants according to the Finnish Personal Data Act 1050/2018.

## 3. Results

Among the MATEX birth cohort, 23,976 singleton births were identified, for which information on maternal and grandmaternal smoking were available. Out of these triads, for 11,848, neither grandmother nor mother were smoking, and for 3668 triads, both grandmother and mother smoked during pregnancy (Table 1).

The average maternal age was 21 years, and the average age of the grandmother when giving birth to the mother was 27 years. Most mothers (75%) were nulliparous. The (grand-)maternal age differed slightly between the smoking categories. In general, the demographics between the mothers and grandmothers when giving birth differed substantially (Table 4).

**Figure 1:**
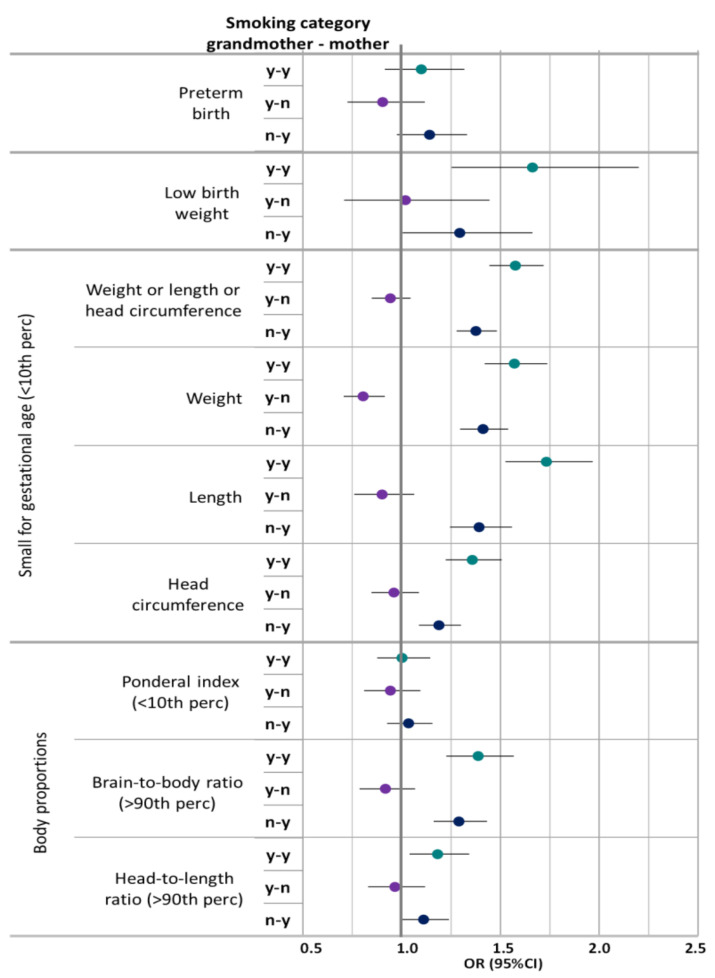
Adjusted ORs for the combined effect of grandmaternal and maternal smoking on birth outcomes in reference to grandmother and mother not smoking. Adjusted for maternal age, sex, maternal parity, maternal socioeconomic status, for grandmaternal age, grandmaternal parity, and grandmaternal socioeconomic status, maternal low birth weight and maternal preterm birth, maternal co-morbidities during pregnancy; low birth weight in addition for gestational age. Smoking categories: y-y: Any smoking grandmother and mother; y-n: Any smoking grandmother, no smoking mother: n-y: No smoking grandmother, any smoking mother; Reference: no smoking grandmother or mother.

Overall, maternal smoking alone was associated with an increased risk for the endpoints considered in this study, albeit not always statistically significantly (Figure 1). The combined effect of grandmaternal and maternal smoking consistently led to higher odds ratios than maternal smoking alone. Grandmaternal smoking alone showed a statistically non-significant association with odds ratios below one for the studied birth outcomes.

Especially, short birth length (OR 1.73, 95%CI: 1.52–1.96) and low birth weight (OR 1.66, 95%CI: 1.25–2.20) were sensitive to the effects of combined grandmaternal and maternal smoking. Preterm birth and ponderal index were not clearly associated with grandmaternal or maternal smoking.

Grandmaternal smoking without maternal smoking was associated with an OR below 1 for low birth weight for gestational age (OR 0.80, 95%CI: 0.70–0.91). In contrast, general low birth weight (<2500 g) did not show a similar association. No association was observed with ponderal index. The ORs for high brain-to-body ratio and high head-to-length ratio were elevated for combined grandmaternal and maternal smoking, as well as only maternal smoking (Figure 1, Table 5, Supplementary Materials Tables S2 and S3). No association was observed with high ponderal index (>90th percentile), low brain-to-body ratio (<10th percentile), or low head-to-length ratio (<10th percentile) (data not shown).

The sensitivity of the ORs to different adjustment models was tested (Table 2). The numerical results changed slightly with different adjustment models. However, the statistical significance remained stable across the models (Table 5, Supplementary Materials, Tables S2 and S3). Between the different adjustment models, the only change in significance was observed for the association between only maternal smoking and head-to-length ratio. The association was not statistically significant for adjustment models M1 and M2, but became significant when including maternal low birth weight, preterm birth and co-morbidities (M3 and M4) (Table 5, Supplementary Materials, Tables S2 and S3).

## 4. Discussion

Smoking during pregnancy is strongly linked with reduced weight and size at birth (for a review see, e.g., [26]). The effect of grandmaternal smoking on size at birth remains discrepant despite an increasing body of research (e.g., [2,3,7]). In this study, we show that when both the grandmother and mother smoked during pregnancy, the effect was stronger than when only the mother smoked. The magnitude of the difference in effect of maternal smoking alone and combined grandmaternal and maternal smoking warrants further studies, especially since the 95% confidence intervals are overlapping indicating astatically non-significant difference.

Research on the adverse effects of grandmaternal smoking is accumulating; however, the effects on birth weight remain inconclusive [2,3]. In addition to birth weight, studies focused on higher body mass index or waist circumference after grandmaternal smoking report positive effects [4,5]. Similar associations have been reported only for the paternal line [6]. The role of sex of the offspring has been studied by Golding and coworkers [7]: They report increased lean body mass in grandsons and reduced body mass in granddaughters after grandmaternal smoking during pregnancy. In addition, increased risk for asthma has been reported after grandmaternal smoking [8,9,10]. Furthermore, autism has been reported to be associated with grandmaternal smoking [11].

The register-based design of this study has the disadvantage that the availability of information is dictated by the register content. In this study, information on smoking in the paternal line, as well as nicotine exposure via means other than tobacco smoke would have been relevant, but are not included in the Finnish Birth Register (MBR). Dougan and colleagues [6] showed that the multigenerational effect of tobacco smoking on obesity may be mediated only via the paternal line. While the evidence for a multigenerational effect is not sufficient yet, the effect of paternal smoking on birth weight has been reported (for a review see [12]). Thus, more research, including the paternal line, is needed and it would be very important to include information of paternal smoking and other nicotine exposure in the MBR. This would facilitate the provision of information to the expectant parents, and not only the mother, about the importance of smoking cessation. Furthermore, it would support the study of the effects of exposure via the paternal line during pregnancy.

In this study, the risk estimates for grandmaternal smoking only (no smoking by the mother) was rather consistently associated with an OR below one, but was only for low birth weight for gestational age statistically significant. This is in line with previous publications in the literature (e.g., [3,27]). Miller and coworkers [3] report an increased birth weight and birth length in grandsons after only grandmaternal smoking during pregnancy. For granddaughters, the association was not statistically significant for birth weight or birth length [3]. It has been proposed that the observed increase in birth weight of grandsons may be due to be residual confounding. Misra and coworkers [27] argue that in their cohort smoking of grandmothers during pregnancy was associated with a higher socioeconomic status and that they may transfer this advantage to their grandchildren. In this work we were able to control for grandmaternal and maternal socioeconomic status. Additionally, the grandmother’s socioeconomic status in our study did not differ from the socioeconomic status of the general Finnish pregnant women. Miller and coworkers [3] propose a direct biological effect of grandmaternal smoking via metabolic programming in maternal somatic cells and/or effects on oocytes during in utero development of the mother. Epigenetic changes after exposure to maternal smoking have been reported in humans (for a review see [28]). Rehan and co-workers report epigenetic changes in testes and ovaries of F2 generation after prenatal and early postnatal nicotine exposure in a rat study [18].

If the observed ORs below 1 for grandmaternal smoking are real and not due to low statistical power or residual confounding, metabolic programming in the maternal line, as proposed by Miller and coworkers [3], may be an underlying mechanism. The inconsistency between the statistically significant OR below 1 for low birth weight for gestational age and the statistically non-significant OR slightly above 1 for low birth weight (<2500 g) suggests that the observed effects are artefacts from low study power or residual confounding.

In this work, the smoking categories available in the MBR of “quitted smoking during first trimester” and “continued smoking after first trimester” have been aggregated to a more general binary variable of “any smoking during pregnancy”. This was necessary to increase the study power with the available cohort. The statistical power of this study differed for each endpoint and exposure group. For the group of newborns, whose grandmother smoked during pregnancy and whose mother did not smoke during pregnancy, the observed ORs were all below the minimum observable OR with 90% study power. However, in comparison with the previously published studies on the effect of grandmaternal smoking, the birth cohort in this work is rather large. Most previous studies were conducted in study populations ranging between several hundreds of triads [2] to 14,000 [4]. The aggregated smoking definition made it impossible to investigate potential differences in the effects between smoking only during early pregnancy and smoking throughout pregnancy.

The birth cohort analyzed in this work has been investigated as part of the bigger MATEX cohort for the effects of maternal smoking [20]. Our previous work included smoking stratified by quitting during the first trimester and continued after the first trimester. Continued maternal smoking was associated with higher risk estimates than quitted smoking. Overall, the results in this work are in line with previously reported results (e.g., [26,29,30]). Our earlier work demonstrated an effect of maternal smoking on body proportions at birth [31]. In this work, grandmaternal smoking was not as clearly associated with changes in body proportions. Interestingly, the proportions are affected in opposite direction, i.e., from high brain-to-body ratio in this work to low brain-to-body ratio in [31]. Thus, body proportions at birth as indicator of sensitivity of different anthropometric indices warrants further studies using maternal and grandmaternal smoking as exposure of interest.

While smoking was the most common route of exposure to nicotine a couple of decades ago, the use of other nicotine products is increasing, both in the treatment for smoking cessation (nicotine replacement therapy), and as lifestyle habits [32]. Nicotine replacement therapy (NRT) is officially recommended to Finnish women, who are unable to quit smoking during pregnancy otherwise [33]. However, there are questions about the safety of NRT during pregnancy [34]. In recent years, the use of e-cigarettes and snus has increased strongly [32,35]. However, there is a lack of studies on the frequency of use among Finnish pregnant women, as well as on the effects on pregnancy and the fetus. However, there is mounting evidence for the adverse effects on the unborn child [36,37]. Considering, that there is increasing amount of evidence that nicotine is the causative agent for at least some adverse effects observed after smoking during pregnancy (for a review, see [34]) it would be extremely important that all exposure to any nicotine-containing products is recorded in the MBR. A big concern is that some young women may switch to long term use of electronic cigarettes or other nicotine products due to the aggressive advertising by the tobacco companies, such as cartoon-based marketing of e-cigarettes [38] and using multiple marketing channels [39]. Using other nicotine products would not be an improvement from the fetal point of view, because nicotine is one of the worst, if not the worst, fetotoxic compound in cigarette smoke [40,41,42].

As to the limitations of the study, the data contained in the MBR register are routinely collected by nurses and midwives during antenatal care visits. They are used regularly in epidemiological research and its data content has been shown to be reliable. Nevertheless, the smoking status is self-reported and thus reporting bias cannot be excluded. Additionally, the MBR does not contain information on the majority of lifestyle factors, such as alcohol consumption, nicotine consumption other than tobacco smoking, diet, and physical exercise. Tobacco smoking status and socioeconomic status are highly correlated with the general lifestyle. They were shown to be reliable makers for unaccounted lifestyle factors [43].

A disadvantage of using register-based data is the unavailability of important confounders [44]. Information on exposure to second hand tobacco smoke of the pregnant women was not available. Importantly, second hand tobacco smoke has been shown to increase the risk for low birth weight and general growth restriction [45]. It cannot be excluded that some part of the effect observed in this study is attributable to potential exposure to second hand tobacco smoke.

Socioeconomic status is an important factor affecting birth outcomes [46,47]. While in this study, the socioeconomic status was not available for about a third of grandmothers and mothers, it has been shown previously that missing socioeconomic status when using MBR data do not bias the results [35]. However, residual confounding cannot be fully excluded.

It has to be noted that the cohort of mothers in this study is not representative of the general population of pregnant women in Finland. The mothers in this study are considerably younger (on average 21 years instead of general pregnant population 29 years) and of lower socioeconomic status than general pregnant women. Furthermore they are more likely to be nulliparous and not married or not in a registered partnership. The general pregnant population in Finland has been described previously [25,36]. These differences are caused by the availability of study population. The study population has been identified from the MBR, which has smoking information with consistent definition available since 1991, thus we could include only mothers born 1991 or later. Considering that the average age at birth of a child was 29 years (25–75th percentiles: 26–33 years) between 1987 and 2015 [25], it becomes clear that in the available cohort, the linkage across three generations leads to a selection of rather young mothers. It is known that younger age at first pregnancy is associated with riskier health behavior in general [48,49,50].

## 5. Conclusions

This study showed that combined grandmaternal and maternal smoking during pregnancy is associated with higher risks for adverse birth outcomes than maternal smoking only. This indicates that there is a transgenerational effect of smoking during pregnancy. The effects of only grandmaternal smoking without any maternal smoking need to be studied in more detail. Further studies are needed to clarify the associations in larger cohorts and to identify underlying mechanisms. It is warranted also to study the effects of nicotine products other than cigarettes, such as nicotine replacement products, snus, and e-cigarettes during pregnancy, as well as exposures mediated via the paternal lineage.

## Figures and Tables

**Table 1:** Smoking during pregnancy in the included triads.

Category	Grandmother	Mother	Frequency	%
n-n	No smoking	No smoking	11,848	49.4
y-y	Any smoking	Any smoking	3668	15.3
y-n	Any smoking	No smoking	2710	11.3
n-y	No smoking	Any smoking	5750	24.0
	**Total**		**23,976**	**100.0**

**Table 2:** Adjustment in regression models *.

Model	Confounder
1	Maternal age, sex, maternal parity, and maternal socioeconomic status
2	Model 1 + grandmaternal age, grandmaternal parity, and grandmaternal socioeconomic status
3	Model 2 + maternal low birth weight and maternal preterm birth
4	Model 3 + maternal co-morbidities during pregnancy

* All regression models for low birth weight were additionally adjusted for gestational age.

**Table 3:** Estimation of the lowest OR detectable as statistically significant (95% CI) with assumed power of 90%.

Exposure Group	Population Size	Exposed (%)	Number Exposed/Number Unexposed	Minimum Detectable OR at Given Endpoint Incidence		
				10%	5%	1%
Any smoking grandmother and any smoking mother	16,000	15	0.333	1.19	1.27	1.66
Any smoking grandmother, no smoking mother	11,000	11	0.225	1.26	1.38	1.97
No smoking grandmother, any smoking mother	17,000	24	0.475	1.15	1.22	1.56

**Table 4:** Description of the study population.

	Mean (sd) or Count (%)			
	No Smoking Grandmother or Mother	Any Smoking Grandmother, any Smoking Mother	Any Smoking Grandmother, no Smoking Mother	No Smoking Grandmother, any Smoking Mother
N (%) of triads	11,848 (49.4)	3668 (15.3)	2710 (11.3)	5750 (24.0)
**Grandmother**				
Age (years)	27.8 (5.6)	25.6 (5.5)	25.9 (5.6)	27 (5.5)
Parity (multiparous)	8215 (69.4%)	2345 (64.1%)	1622 (59.9%)	3918 (68.2%)
Marital status (married, registered partnership)	9031 (76.4%)	1463 (40.1%)	1268 (47%)	3739 (65.3%)
*Socioeconomic status*				
Upper white collar	766 (6.5%)	77 (2.1%)	64 (2.4%)	249 (4.3%)
Lower white collar	5298 (44.7%)	1168 (31.8%)	945 (34.9%)	2324 (40.4%)
Blue collar	2624 (22.1%)	1276 (34.8%)	956 (35.3%)	1607 (27.9%)
Other socioeconomic status (e.g., student, farmer, self-employed)	2721 (23%)	903 (24.6%)	576 (21.3%)	1282 (22.3%)
No information on socioeconomic status	439 (3.7%)	244 (6.7%)	169 (6.2%)	288 (5%)
**Mother**				
Age (years)	21.4 (2)	20.3 (2.1)	21.1 (2.2)	20.5 (2)
Height (cm)	165 (6)	164 (5.9)	164.2 (6)	164.9 (5.9)
Weight (kg)	64.4 (14)	65.5 (15.6)	66.2 (15.2)	64.9 (14.9)
Parity (multiparous)	3767 (31.8%)	871 (23.8%)	839 (31%)	1156 (20.1%)
Marital status (married, registered partnership)	4479 (37.8%)	386 (10.5%)	560 (20.7%)	867 (15.1%)
*Socioeconomic status*				
Upper white collar	83 (0.7%)	8 (0.2%)	12 (0.4%)	25 (0.4%)
Lower white collar	1700 (14.3%)	257 (7%)	309 (11.4%)	554 (9.6%)
Blue collar	1006 (8.5%)	287 (7.8%)	188 (6.9%)	577 (10%)
Other socioeconomic status (e.g., student, farmer, self-employed)	2302 (19.4%)	869 (23.7%)	521 (19.2%)	1340 (23.3%)
No information on socioeconomic status	6757 (57%)	2247 (61.3%)	1680 (62%)	3254 (56.6%)
Birth weight (g)	3588.5 (504.3)	3365.6 (489.4)	3368.8 (496.7)	3585.7 (506)
Birth length (cm)	50.2 (2.1)	49.3 (2.1)	49.3 (2.2)	50.1 (2.1)
Gestational age (weeks)	39.6 (1.5)	39.4 (1.5)	39.5 (1.6)	39.6 (1.5)
Co-morbidities (yes)	1435 (12.1%)	515 (14%)	406 (15%)	702 (12.2%)
**Child**				
Gestational age (weeks)	39.4 (1.7)	39.4 (1.9)	39.5 (1.6)	39.4 (1.8)
Birth weight (g)	3509.7 (510.3)	3376.7 (543.9)	3528.9 (496.8)	3416 (530.8)
Birth length (cm)	50 (2.3)	49.4 (2.5)	50.1 (2.3)	49.6 (2.5)
Head circumference (cm)	34.8 (1.5)	34.5 (1.7)	34.8 (1.5)	34.6 (1.7)
Sex (male)	6104 (51.5%)	1833 (50%)	1404 (51.8%)	2968 (51.6%)
PTB (yes)	511 (4.3%)	194 (5.3%)	115 (4.2%)	290 (5%)
LBW (yes)	325 (2.8%)	169 (4.7%)	73 (2.7%)	213 (3.8%)
Small for gestational age (<10th percentile) ^#^ (yes)	2805 (23.7%)	1285 (35%)	635 (23.4%)	1877 (32.6%)
Small weight for gestational age (<10th percentile) (yes)	1710 (14.4%)	844 (23%)	337 (12.4%)	1223 (21.3%)
Small length for gestational age (<10th percentile) (yes)	883 (7.5%)	486 (13.3%)	188 (6.9%)	621 (10.8%)
Small head circumference for gestational age (<10th percentile) (yes)	1660 (14.1%)	729 (20.1%)	384 (14.3%)	1046 (18.4%)
Low ponderal index (<10th percentile) (yes)	1098 (10.3%)	359 (10.8%)	243 (10%)	583 (11.3%)
High head-to-length ratio (>90th percentile) (yes)	1120 (10.6%)	490 (14.9%)	237 (10%)	723 (14.1%)

^#^ birth weight, birth length, or head circumference <10th percentile.

**Table 5:** Adjusted odds ratios for the effect of 2-generational smoking during pregnancy on birth outcomes in reference to no smoking during pregnancy of grandmother and mother.

	OR (95%CI)					
	Model 3 *			Model 4 ^		
Outcome	Any Smoking Mother and Grandmother	Any Smoking Grandmother and no Smoking Mother	No Smoking Grandmother and any Smoking Mother	Any Smoking Mother and Grandmother	Any Smoking Grandmother and no Smoking Mother	No Smoking Grandmother and any Smoking Mother
**Preterm birth**	1.129 (0.94–1.351)	0.944 (0.759–1.163)	1.134 (0.971–1.323)	1.1 (0.915–1.317)	0.904 (0.726–1.117)	1.141 (0.976–1.331)
**Low birth weight**	1.658 (1.249–2.193)	1.02 (0.709–1.446)	1.292 (1.004–1.66)	1.662 (1.253–2.2)	1.019 (0.709–1.445)	1.294 (1.005–1.662)
**Small for gestational age**						
Birth weight(<10th percentile))	1.572 (1.421–1.738)	0.807 (0.707–0.917)	1.412 (1.296–1.539)	1.571 (1.42–1.737)	0.805 (0.706–0.915)	1.412 (1.296–1.539)
Birth length (<10th percentile)	1.727 (1.52–1.96)	0.896 (0.756–1.058)	1.393 (1.244–1.558)	1.733 (1.525–1.967)	0.901 (0.76–1.064)	1.392 (1.244–1.558)
Head circumference (<10th percentile)	1.351 (1.217–1.499)	0.953 (0.841–1.079)	1.19 (1.088–1.301)	1.358 (1.223–1.507)	0.961 (0.847–1.087)	1.189 (1.087–1.3)
Any of the above^#^ (<10th percentile)	1.57 (1.438–1.713)	0.938 (0.845–1.04)	1.376 (1.278–1.482)	1.576 (1.444–1.719)	0.943 (0.849–1.045)	1.376 (1.278–1.482)
**Body proportions**						
Ponderal index (<10th percentile)	1.003 (0.877–1.146)	0.947 (0.813–1.099)	1.035 (0.925–1.156)	1.002 (0.876–1.145)	0.943 (0.8100–1.095)	1.035 (0.926–1.156)
Brain-to-body ratio (>90th percentile)	1.395 (1.233–1.576)	0.927 (0.794–1.077)	1.288 (1.16–1.43)	1.387 (1.226–1.568)	0.918 (0.787–1.068)	1.29 (1.162–1.432)
Head-to-length ratio (>90th percentile)	1.176 (1.03–1.342)	0.972 (0.833–1.131)	1.126 (1.007–1.259)	1.176 (1.029–1.341)	0.969 (0.83–1.127)	1.127 (1.007–1.259)

* adjusted as Model 2 (see Table 2) and additionally for maternal low birth weight and maternal preterm birth. ^ adjusted for Model 3 and in addition for maternal co-morbidities during pregnancy. ^#^ birth weight, birth length, or head circumference <10th percentile.

## Data Availability

Data may be obtained from a third party and are not publicly available. The Finnish Institute of Health and Welfare is the controller of the Medical Birth Register. Data may be obtained from the register controller (https://thl.fi/en/web/thlfi-en/statistics/information-on-statistics/register-descriptions/newborns, accessed on 8 March 2021).

## Supplementary Materials

The following are available online at https://www.mdpi.com/article/10.3390/ijerph18094985/s1. Table S1. Endpoint definitions. Table S2. Unadjusted Odds ratios for the effect of grandmaternal and maternal smoking during pregnancy on birth outcomes in reference to no smoking during pregnancy of grandmother and mother. Table S3. Adjusted Odds ratios for the effect of grandmaternal and maternal smoking during pregnancy on birth outcomes in reference to no smoking during pregnancy of grandmother and mother (Adjustment models 1 and 2).
