# Supplementary material for: Effect of Grandmaternal Smoking on Body Size and Proportions at Birth
Source: Int J Environ Res Public Health. 2021 May 7;18(9):4985. doi: 10.3390/ijerph18094985 (PMC8124860; doi:10.3390/ijerph18094985)
Supplement: Supplementary file 1 [file ijerph-18-04985-s001.zip › ijerph-1161242-supplementary.pdf]

# Supplemental Material

## Effect of grandmaternal smoking on body size and proportions at birth

**Isabell K. Rumrich [1*], Otto Hänninen [1], Matti Viluksela [1,2], Kirsi Vähäkangas [2]**

[1] Finnish Institute for Health and Welfare (THL), Department of Health Security, P.O. Box 95, 70701 Kuopio, Finland

[2] University of Eastern Finland (UEF), Faculty of Health Sciences, School of Pharmacy/Toxicology, P.O. Box 1627, 70211 Kuopio, Finland

* Correspondence: isabell.rumrich@thl.fi; Finnish Institute for Health and Welfare (THL), Department of Public Health Solutions, P.O. Box 95, 70701 Kuopio, Finland; telephone: +358 29 524 7030

Many long-term adverse effects of smoking during pregnancy are known. Increasingly, adverse effects in the grandchild after grandmaternal smoking during pregnancy are reported. We explored this in a birth cohort of 24,000 grandmother-mother-child triads identified from the Finnish Medical Birth Register in 1991-2016. Multiple logistic regression was used to analyse the association between any smoking during pregnancy by both grandmother and mother, or only grandmother or mother on adverse birth outcomes. No smoking by neither grandmother nor mother was used as the reference. As endpoints, preterm birth, low birth weight, small for gestational age (birth weight, birth length, head circumference) and body proportionality (low ponderal index, high brain-to-body ratio, high head-to-length ratio) were included. Smoking by both grandmother and mother was consistently associated with higher risks than smoking only by the mother. Birth length and weight were especially sensitive to (grand)maternal smoking. In conclusion, the combined effect of grandmaternal and maternal smoking is stronger than the effect ofassociated with higher risks than only maternal smoking.

**Keywords**: pregnancy, tobacco, smoking, maternal, grandmaternal, birth outcome, birth weight, register research, intergenerational

**Table S1. Endpoint definitions**

| Endpoint | | Definition case | Definition control |
|---|---|---|---|
| 1 | Preterm birth | Gestational age <37 weeks | Gestational age ≥37 weeks |
| 2 | Low birth weight | Birth weight <2500 g | Birth weight 2,500-4,500 g |
| 3 | Small for gestational age (based on Finnish reference growth charts) (Sankilampi et al. 2013) | | |
| | Any | <10th percentile of weight and/or length and/or head circumference for gestational age (weeks) birth | ≥10th percentile of weight and length and head circumference for gestational age (weeks) birth |
| | Weight | <10th percentile of weight for gestational age (weeks) birth | ≥10th percentile of weight for gestational age (weeks) birth |
| | Body length | <10th percentile of length for gestational age (weeks) birth | ≥10th percentile of length for gestational age (weeks) birth |
| | Head circumference | <10th percentile of head circumference for gestational age (weeks) birth | ≥10th percentile of head circumference for gestational age (weeks) birth |
| 4 | Body proportions | | |
| | Ponderal index | <10th percentile of weight-to-length ratio for gestational age (weeks) birth | 10-90th percentile of weight-to-length ratio for gestational age (weeks) birth |
| | Brain-to-body ratio [a] | >90th percentile of weight-to-head circumference ratio for gestational age (weeks) birth | 10-90th percentile of weight-to-head circumference ratio for gestational age (weeks) birth |
| | Head-to-length ratio [a] | >90th percentile of head circumference-to-length ratio for gestational age (weeks) birth | 10-90th percentile of head circumference-to-length ratio for gestational age (weeks) birth |

[a] available for the years 2004-2016

**Table S2. Unadjusted Odds ratios for the effect of grandmaternal and maternal smoking during pregnancy on birth outcomes in reference to no smoking during pregnancy of grandmother and mother**

| Outcome | OR (95%CI) | | |
|---|---|---|---|
| | Any smoking mother & grandmother | Any smoking grandmother & no smoking mother | No smoking grandmother & any smoking mother |
| **Preterm birth** | 1.239 (1.044-1.465) | 0.983 (0.796-1.204) | 1.178 (1.016-1.365) |
| **Low birth weight** | 1.702 (1.405-2.054) | 0.978 (0.751-1.258) | 1.357 (1.137-1.616) |
| **Small for gestational age (<10th percentile)** | | | |
| **Birth weight** | 1.772 (1.615-1.943) | 0.842 (0.742-0.953) | 1.603 (1.477-1.738) |
| **Birth length** | 1.896 (1.685-2.132) | 0.926 (0.784-1.087) | 1.504 (1.35-1.675) |
| **Head circumference** | 1.523 (1.383-1.677) | 1.013 (0.898-1.141) | 1.365 (1.254-1.486) |
| **Any of the above #** | 1.738 (1.605-1.883) | 0.987 (0.894-1.088) | 1.562 (1.457-1.675) |
| **Body proportions** | | | |
| **Ponderal index (<10th percentile)** | 1.054 (0.928-1.195) | 0.979 (0.836-1.121) | 1.105 (0.993-1.228) |
| **Brain-to-body ratio (>90th percentile)** | 1.467 (1.308-1.644) | 0.931 (0.801-1.077) | 1.373 (1.242-1.518) |
| **Head-to-length ratio (>90th percentile)** | 1.196 (1.057-1.352) | 0.962 (0.827-1.114) | 1.142 (1.026-1.27) |

# birth weight, birth length or head circumference <10th percentile

Statistically significant (p<0.05) associations highlighted in light red background.

**Table S3. Adjusted Odds ratios for the effect of grandmaternal and maternal smoking during pregnancy on birth outcomes in reference to no smoking during pregnancy of grandmother and mother**

| | OR (95%CI) | | | | | |
|---|---|---|---|---|---|---|
| | Model 1* | | | Model 2^ | | |
| Outcome | Any smoking mother & grandmother | Any smoking grandmother & no smoking mother | No smoking grandmother & any smoking mother | Any smoking mother & grandmother | Any smoking grandmother & no smoking mother | No smoking grandmother & any smoking mother |
| **Preterm birth** | 1.19 (0.997-1.416) | 0.978 (0.791-1.199) | 1.138 (0.977-1.323) | 1.154 (0.963-1.379) | 0.963 (0.776-1.184) | 1.134 (0.972-1.319) |
| **Low birth weight** | 1.671 (1.274-2.184) | 1.003 (0.705-1.406) | 1.277 (0.997-1.632) | 1.718 (1.299-2.266) | 1.035 (0.721-1.464) | 1.279 (0.997-1.636) |
| **Small for gestational age (<10th percentile)** | | | | | | |
| **Birth weight** | 1.618 (1.468-1.783) | 0.823 (0.724-0.934) | 1.415 (1.3-1.54) | 1.629 (1.474-1.8) | 0.825 (0.724-0.938) | 1.411 (1.296-1.536) |
| **Birth length** | 1.778 (1.572-2.008) | 0.924 (0.782-1.087) | 1.391 (1.244-1.554) | 1.784 (1.572-2.023) | 0.919 (0.776-1.084) | 1.389 (1.242-1.552) |
| **Head circumference** | 1.414 (1.277-1.563) | 1.000 (0.884-1.128) | 1.217 (1.115-1.329) | 1.381 (1.245-1.531) | 0.971 (0.857-1.098) | 1.206 (1.104-1.317) |
| **Any of the above #** | 1.623 (1.492-1.766) | 0.973 (0.879-1.076) | 1.394 (1.296-1.499) | 1.612 (1.478-1.758) | 0.962 (0.868-1.066) | 1.389 (1.291-1.493) |
| **Body proportions** | | | | | | |
| **Ponderal index (<10th percentile)** | 1.009 (0.885-1.149) | 0.957 (0.824-1.107) | 1.038 (0.9300-1.158) | 1.022 (0.894-1.167)) | 0.959 (0.824-1.113) | 1.036 (0.928-1.156) |
| **Brain-to-body ratio (>90th percentile)** | 1.408 (1.25-1.585) | 0.928 (0.798-1.075) | 1.276 (1.151-1.414) | 1.424 (1.26-1.608) | 0.939 (0.805-1.09) | 1.285 (1.158-1.425) |
| **Head-to-length ratio (>90th percentile)** | 1.182 (1.04-1.342) | 0.966 (0.83-1.119) | 1.111 (0.995-1.239) | 1.164 (1.02-1.325) | 0.963 (0.826-1.119) | 1.113 (0.997-1.242) |

* adjusted for maternal age, sex, maternal parity, maternal socioeconomic status; low birth weight in addition for gestational age

^ adjusted as Model 1 and additionally for grandmaternal age, grandmaternal parity, and grandmaternal socioeconomic status

# birth weight, birth length or head circumference <10th percentile

Statistically significant (p<0.05) associations highlighted in light red background
